# Madelung’s disease and pulmonary aspergillosis: a case report and literature review

**DOI:** 10.1186/s13019-020-01166-3

**Published:** 2020-05-25

**Authors:** Weijiang Ma, Xilong Zhao, Xu Li, Chenxi Zang, Limin Yang, Yan Wang, Xin Liu

**Affiliations:** 1 Department of Thoracic Surgery, Second Affiliated Hospital of Kunming Medical University, 374th Dianmian Road, Kunming, 650101 China; 2 Department of Pathology, 920th Hospital of Joint Logistics Support Force of Chinese People’s Liberation Army, Kunming, 652230 China; 3 Department of Pathology, Second Affiliated Hospital of Kunming Medical University, Kunming, 650101 China

**Keywords:** Madelung’s disease, Pulmonary aspergillosis, Opportunistic pathogen, Immune disorder

## Abstract

**Background:**

Madelung’s disease (MD) is a rare disorder of fat metabolism, which is usually associated with diabetes, hyperuricemia, liver disease, nevertheless there is no report of a patient with MD and pulmonary aspergillosis (PA). This article aimed to enhance the awareness of this two diseases and discuss the possible mechanism of the combination of them preliminarily.

**Case presentation:**

In this case, we described a 56-year-old male patient with cough, expectoration and dyspnea. His neck has a very peculiar appearance. Chest enhanced CT scan showed there were multiple nodules in both lungs, some of which had cavities and the mediastinal lymph nodes were swollen. Ultrasound scan of the neck showed diffuse hyperplasia of subcutaneous fat in neck and bilateral supraclavicular fossa. Fortunately, after performing pulmonary wedge resection aimed at pathological examination and giving relevant treatments, this patient was finally diagnosed as MD with PA, and his symptoms were significantly relieved.

**Conclusions:**

MD is rare, the phenomenon that MD combined with PA is rarer. Immune disorder may be the possible mechanism.

## Background

Multiple symmetric lipomatosis (MSL), also called Madelung’s disease (MD), is a rare disorder of fat metabolism, which is characterized by progressive, symmetric deposition of non-enveloped adipose tissue between superficial and deep fascia in typical locations [1]. Pulmonary aspergillosis (PA) is caused by a variety of *Aspergilli* which is a king of opportunistic pathogen. MD combined with diabetes, hyperuricemia and liver disease is common, nevertheless, there is no report of a patient with MD and PA. Here, we report a case of 56-year-old male patient with MD and PA, and discuss the possible mechanism.

## Case presentation

**Fig. 1:**
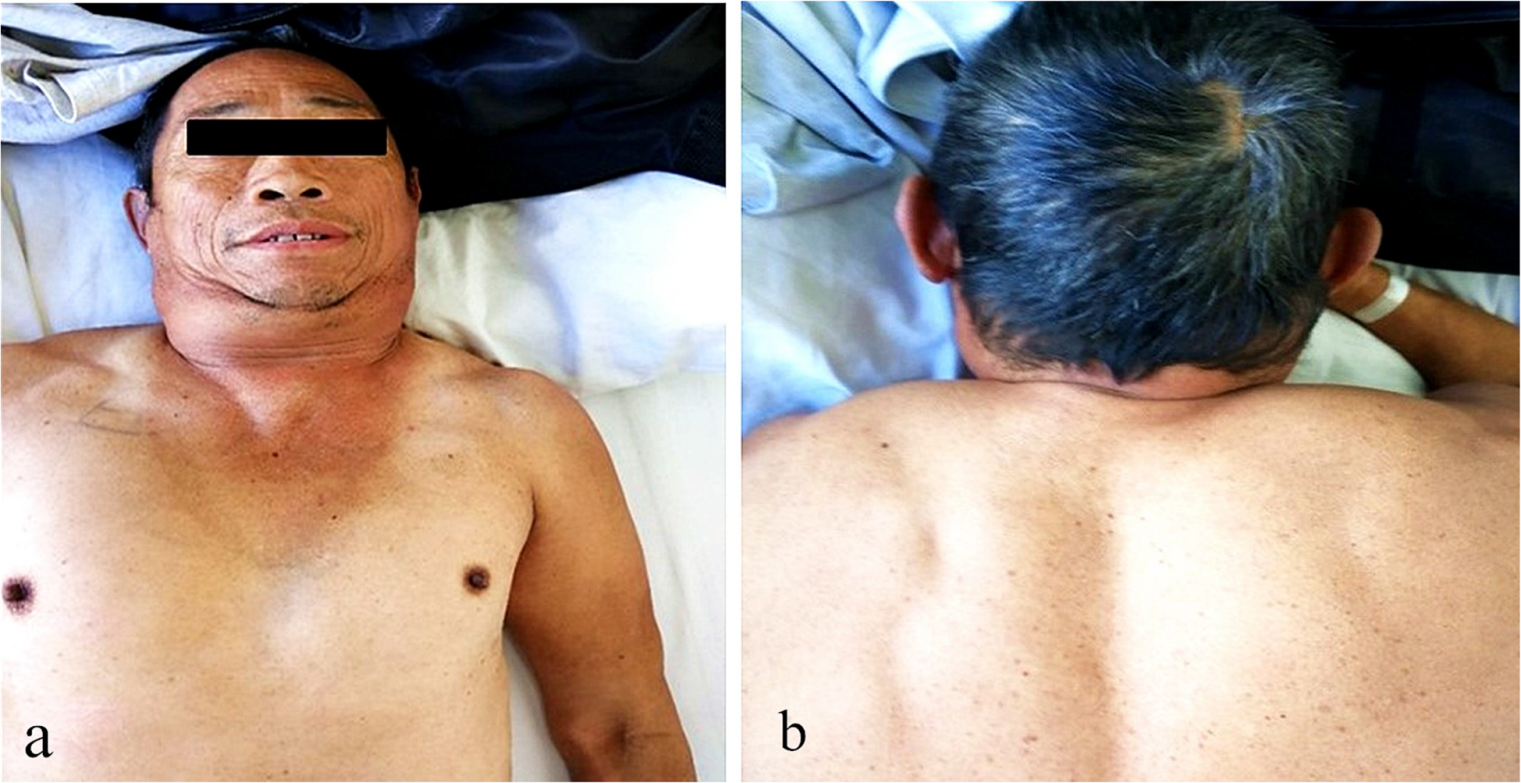
Symmetric swellings were observed on neck, back and upper chest. **a** Front view. **b** Back view

**Fig. 2:**
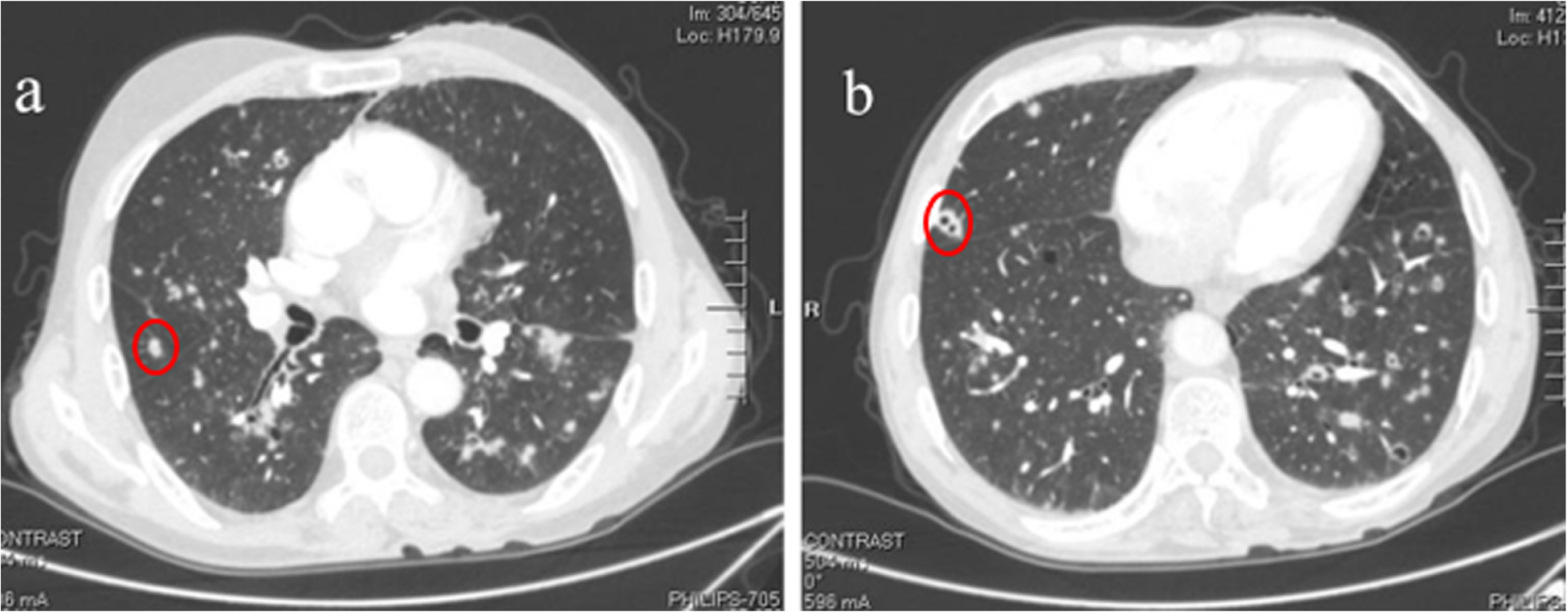
Chest enhanced CT:multiple nodules in both lungs, some of which had cavities. **a** The first lesion we resected. **b** The second lesion we resected

**Fig. 3:**
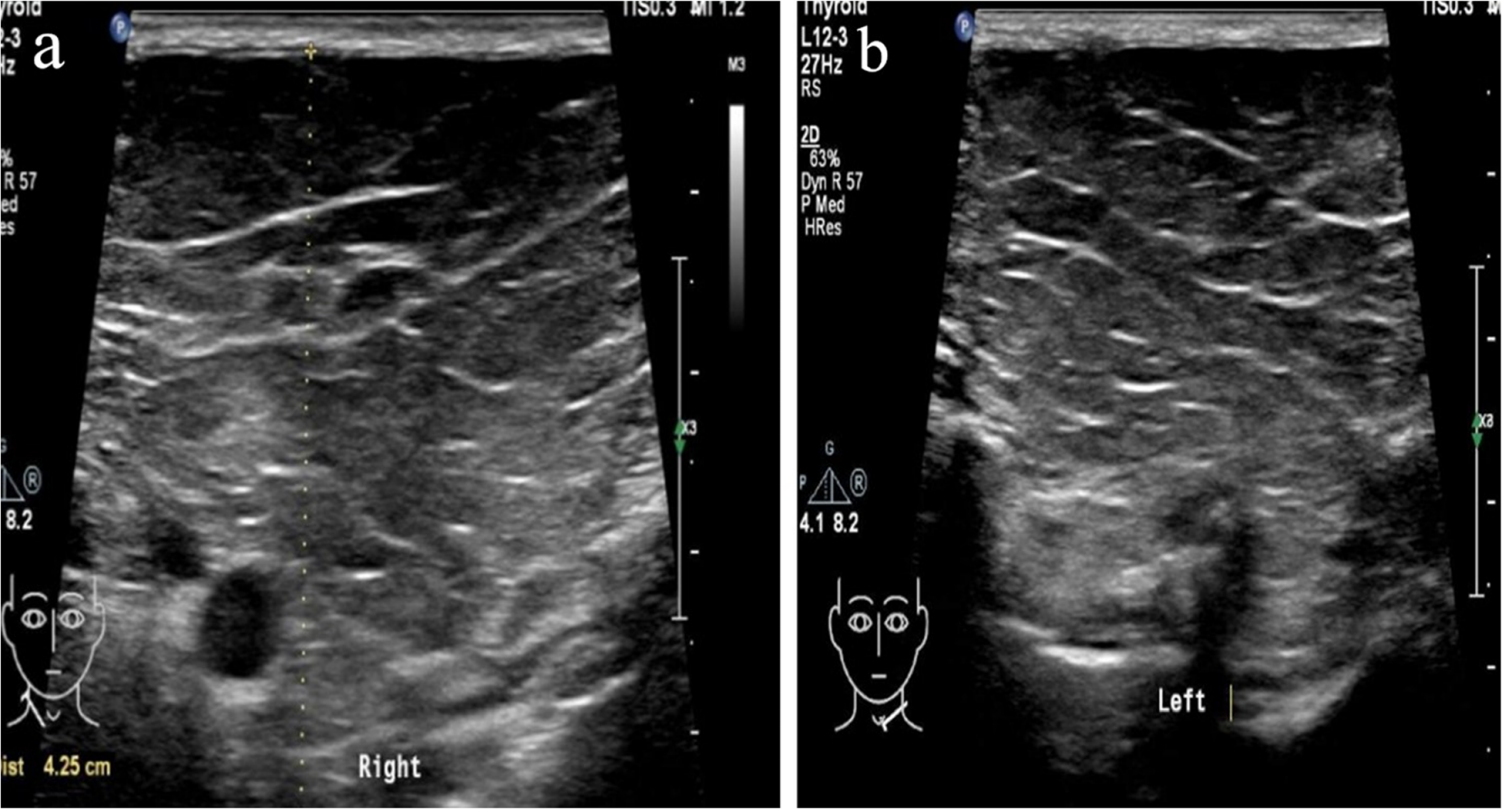
Ultrasound scan of the neck: diffuse hyperplasia of subcutaneous fat in neck and bilateral supraclavicular fossa, the thickest is 4.3 cm on the right and 4.5 cm on the left. **a** Right. **b** Left

**Fig. 4:**
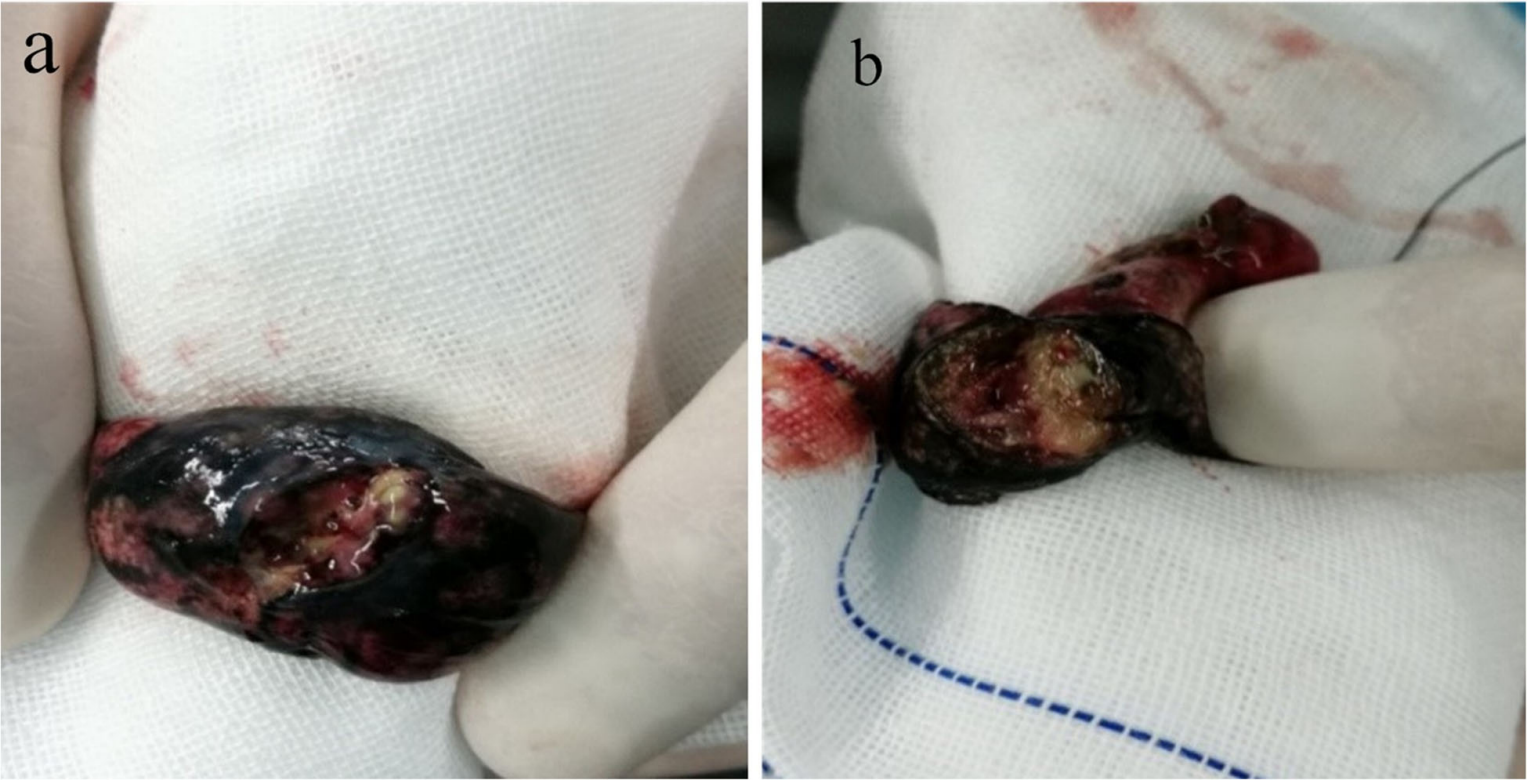
Macroscopic observation of two typical lesions. **a** Nodule 1 **b** Nodule 2

**Fig. 5:**
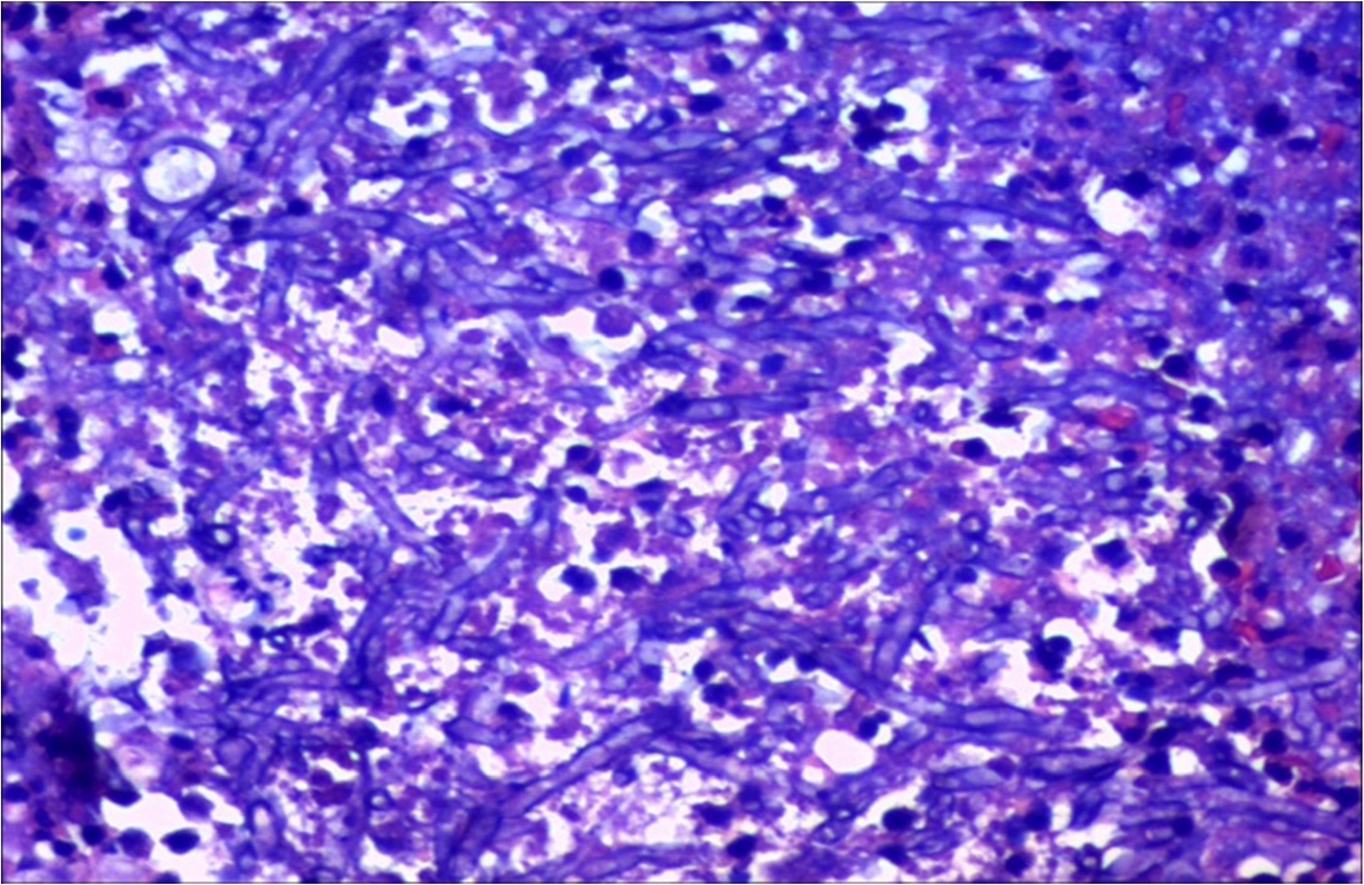
Paraffin section of lesions: A large number of inflammatory cells infiltrated, fungal hyphae could be seen, fungal fluorescence staining(+)

A 56-year-old male patient was presented to our hospital due to cough, expectoration and dyspnea for more than half a year. Chest CT examination in the local hospital showed that there were multiple cavitary shadows in bilateral lungs. However, there was no significant remission after antibiotic administration. For further diagnosis and treatment, he came to our hospital. This patient had a 30-year personal history of smoking and a 38-year history of heavy drinking. Physical examination: temperature 36 °C, heart rate 70 beats per minute, blood pressure 103/62 mmHg, oxygen saturation 92% (without oxygen inhalation), body mass index (BMI) was 19.43 kg/m^2^. Symmetric swellings were observed on neck, back and upper chest, which were painless, soft and had clear boundary (Fig. 1). Breath sound of both lower lung fields was weakened slightly and there was a little wet rale. Chest enhanced CT scan showed there were multiple nodules in both lungs, some of which had cavities and the mediastinal lymph nodes were swollen (Fig. 2). Ultrasound scan of the neck showed diffuse hyperplasia of subcutaneous fat in neck and bilateral supraclavicular fossa (Fig. 3). Ultrasound scan of the thyroid was normal. Laboratory testing showed as follows: ALT 63 U/L (normal range: 5–40), AST 48 U/L (normal range: 8–40), total cholesterol 4.01 mmol/L (normal range:3.49–5.18), triglyceride 1.03 mmol/L (normal range:0.25–1.71), hypersensitive C-reactive protein 65.3 mg/L (normal range:< 10),antinuclear antibody (ANA) was positive, serum IgG 16.2 g/L (normal range:7–16), galactomannan was normal, however the fungal 1,3-β-D-glucan was 357.6 pg/ml (normal range:< 100). Lung biopsy was performed under CT guidance, however, pathological examination showed chronic inflammation only. To clarify the pulmonary niduses, we decided to operate pulmonary wedge resection aimed at that two typical niduses (Fig. 2). After resecting lung tissues containing the niduses, the lung tissues were immediately dissected and observed, we found the nodules were offwhite and necrotic by naked eyes (Fig. 4). The pathological examination after operation showed pulmonary aspergillosis (Fig. 5). The patient recovered and left the hospital after antifungal therapy (Voriconazole, on the first day of treatment, 300 mg each time, twice a day, and then 200 mg each time, twice a day, intravenous drip, lasting for 6 weeks). The patient was diagnosed as pulmonary aspergillosis with Madelung’s disease finally.

## Discussion

MD is a rare disease that was firstly reported by Brodie in 1846 with higher incidence in middle-aged and old men [2]. In the Mediterranean and Eastern Europe, the incidence of MD is more frequent in Caucasian and about 15:1 between men and women. At present, about 400 cases are reported in the world [3]. Two common types of this disease can be observed, according to the distribution of lipomatosis. Type I occurs in men commonly, which is characterized by an accumulation of fat around the neck, back, shoulder and upper arm, we named it the “bull’s neck, the horse’s collar and the hump sign”. Type II is similar to obesity, which is characterized by an accumulation of fat around the deltoid, upper back, hip and thigh, and there is no significant difference in the incidence of this type between men and women [4]. In addition, children with congenital MD that caused deformity of skeletons were classified as type III by Parmar [5]. Considering clinical manifestations and auxiliary examinations, this case was diagnosed as type I.

Alcohol is a risk factor for MD, about 95% of patients have a long heavy drinking history [6]. Besides, MD is also associated with diabetes, hyperuricemia, liver disease, hypothyroidism, peripheral nerve damage*,* etc. [7]. The patient had a drinking history for 38 years. At present, the pathogenesis of MD is not completely clear, the possible mechanism of fat accumulation is the decrease of β-adrenergic receptor quantities and functions and the mutation of mitochondrial DNA (mtDNA) caused by long-term drinking [8, 9], and 8344A → G is the main mutation site [10]. For the treatments of MD, there are no special treatments, it mainly includes smoking cessation, alcohol abstinence, weight control, drug treatment and surgical resection for hyperplastic fat [11]. Compared with conservative treatments, surgical operations are preferred for MD, and the effect is more definite, including liposuction and lipectomy. Liposuction is suitable for patients with small fat volume, while lipectomy is the opposite, however, both of them are difficult to achieve complete excision for fat and patients often relapse after treatment. Brea [7] reported postoperative recurrence rate following lipectomy, liposuction, and lipectomy combined with liposuction were 51, 95, and 50%, respectively.

In recent years, due to the nonstandard application of antibiotics, immunosuppressants and the variation of strains, the incidence of PA has increased year by year. PA is caused by *Aspergillus* infection in lung tissue. Though *Aspergillus* is widely distributed in nature, including air, soil, water, moldy food, etc., only a few have the ability to cause disease, *Aspergillus fumigatus* is the main pathogenic strain [12]. Aspergillus also exists in the normal respiratory tract, it does not lead to infection when the immune function of the body is normal, nevertheless, it’s able to cause opportunistic infection when immune system is disordered. The common causes include long-term use of immunosuppressants, blood system diseases, advanced tumor cachexia, AIDS, severe diabetes, etc. [13]. Due to the lack of specificity symptoms, PA is difficult to diagnose. The “gold standard” of confirmative diagnosis for PA can be aided by the combination of histopathologic/cytologic and culture specimens. However, due to the difficulty in obtaining tissue samples and the long time of culture, there are some limitations in “gold standard”, which makes it difficult to diagnose early and timely. CT plays a vital role in diagnosis for PA, it commonly shows multiple nodules, consolidation, cavities, stars and moon sign, halo sign, etc.

The patient came to our hospital because of “cough, expectoration and dyspnea more than half a year”. We found the abnormal enlargement on the neck by physical examination, so we think that the dyspnea was caused by the compression of the respiratory tract by the swellings. However, “with the swellings on neck for more than a decade, I didn’t have dyspnea until half a year ago”, this patient said. We have to think about other reasons. In view of CT and fungal 1,3 - β - D-glucan, we taken the possibility of fungal infection into consideration. In order to clarify the diagnosis, lung biopsy was performed under CT guidance, but the pathological examination didn’t show clear evidence. Next, we decided to resect the two typical lesions for obtaining the tissue for pathological examination again, and PA was diagnosed in the end.

It’s more common that MD is associated with diabetes, hyperuricemia and peripheral nerve damage, nevertheless, there are no reports about the case with MD and PA. Thus, we propose to discuss the possible mechanism of MD and PA in this case. MD is a metabolic disorder disease which is accompanied by gene changes. In this case, ANA was positive and IgG was abnormal, thus we put forward the hypothesis that apart from the disorder of fat metabolism, MD would be able to affect the immune function of the body and cause immune disorder, which leads to aspergillosis infection more easily. But this is only a case, it could just be a coincidence, if we need to further verify the hypothesis, we need more similar cases, and detect the immune evaluation indicators such as serum immunoglobulin, CD4^+^ T cells and CD8^+^T cells, and carry out the corresponding statistical analysis for in-depth study. Besides, this patient had a 30-year personal history of smoking, we should keep in mind that chronic smoking is a potential risk factor for pulmonary aspergillosis, because nicotine, tar, carbon monoxide and other harmful substances produced by cigarette combustion can cause respiratory tract inflammation, increase mucus secretion, inhibit ciliary movement and reduce purification function of respiratory tract [14], which would make it easier for pathogens to deposit in the lungs.

## Conclusions

MD is rare, the phenomenon that MD combined with PA is rarer. Immune disorder may be the possible mechanism.

## Data Availability

The datasets used and/or analyzed in the current article are available from the corresponding author on reasonable request.

## Abbreviations

MD: Madelung’s disease
PA: Pulmonary aspergillosis
MSL: Multiple symmetric lipomatosis
BMI: body mass index
ANA: Antinuclear antibody
mtDNA: mitochondrial DNA
